# Preoperative Serum Tumor Marker Levels in Gastric Cancer

**DOI:** 10.12669/pjms.301.3968

**Published:** 2014

**Authors:** Erdal Polat, Ugur Duman, Mustafa Duman, Kivanc Derya Peker, Cebrail Akyuz, Necdet Fatih Yasar, Orhan Uzun, Sabiye Akbulut, Erdal Birol Bostanci, Sinan Yol

**Affiliations:** 1 Erdal Polat, MD, Department of Gastrointestinal Surgery, Kosuyolu High Specialty Training and Research Hospital, Istanbul, Turkey.; 2 Ugur Duman, MD, Bursa Sevket Yilmaz Training and Research Hospital, Department of General Surgery, Bursa, Turkey.; 3 Mustafa Duman, MD, Associate Professor, Department of Gastrointestinal Surgery, Kosuyolu High Specialty Training and Research Hospital, Istanbul, Turkey.; 4 Kivanc Derya Peker, MD, Department of Gastrointestinal Surgery, Kosuyolu High Specialty Training and Research Hospital, Istanbul, Turkey.; 5 Cebrail Akyuz, MD, Department of Gastrointestinal Surgery, Kosuyolu High Specialty Training and Research Hospital, Istanbul, Turkey.; 6 Necdet Fatih Yasar, MD, Department of Gastrointestinal Surgery, Kosuyolu High Specialty Training and Research Hospital, Istanbul, Turkey.; 7 Orhan Uzun, MD, Department of Gastrointestinal Surgery, Kosuyolu High Specialty Training and Research Hospital, Istanbul, Turkey.; 8 Sabiye Akbulut, MD, Department of Gastroenterology, Kosuyolu High Specialty Training and Research Hospital, Istanbul, Turkey.; 9 Erdal Birol Bostanci, MD, Professor, Department of Gastrointestinal Surgery, Turkiye High Specialty Training and Research Hospital, Ankara, Turkey.; 10 Sinan Yol, MD, Professor, Department of Gastrointestinal Surgery, Kosuyolu High Specialty Training and Research Hospital, Istanbul, Turkey.

**Keywords:** Gastric cancer, Peritoneal carcinomatozis, Serum tumor markers

## Abstract

***Objective:*** Tumor markers have shown little benefit as a method for screening. However, they can be used clinically for the monitoring of tumor recurrence and used as prognostic factors because higher levels have been observed in advanced disease. This study aimed to investigate the relationship between the preoperative tumor marker levels and different clinical aspects of gastric cancer.

***Methods:*** One hundred and six consecutive patients with confirmed diagnosis of gastric cancer and 106 subjects (age and sex matched) with no malignancy as control group were included prospectively in this study in 3 years. The relationships between tumor markers CEA, CA 19-9 and stage of disease, tumor differentiation, presence of ringlet cell type, presence of peritoneal carcinomatozis were investigated.

***Results:*** The serum CEA and CA 125 levels were found to be significantly elevated in gastric cancer patients than in controls. The serum level of CEA had showed a significant elevation with the presence of distant metastasis. The CA 19-9 and CA 125 levels had showed significant elevations with the presence of peritoneal carcinomatozis.

***Conclusions***: This study showed that there is a limited clinical benefit of preoperative tumor marker measurements in gastric cancer such as estimation of peritoneal dissemination.

## INTRODUCTION

Gastric cancer remains one of the most common causes of cancer related death worldwide and the predicted incidence for 2010 is over one million as expected in previous reports. Gastric malignancies also show extensive tumor invasion and early spread to either regional lymph nodes or distant sites^1^^-^^4^ and this aggressive stance gives rise to the challenging questions about the way of the preoperative management strategies.

The stage oriented management of gastric cancer is one of the recent proposals and it is crucial to improve the outcome of patients with operable gastric cancer.^5^^-^^7^ Currently, preoperative staging relies on imaging studies. None of the available imaging modalities is sufficient to reliably confirm the presence or the number of regional lymph node metastasis and are inefficient to guide a clinical decision depending on nodal status.^8^

Carcinoembryonic antigen (CEA) and carbohydrate antigen 19-9 (CA 19-9) are commonly used markers for gastric cancer.^9^ Although both markers are often measured in patients with gastric cancer preoperatively, the clinical correlation between CEA and CA 19-9 is reported as unclear and controversial.^10^^-^^12^ In addition to these common used markers; carbohydrate antigen 125 (CA 125) and carbohydrate antigen 72-4 (CA 72-4) have been reported to be elevated in advanced gastric cancer.^13^

Tumor markers have shown little benefit as a method for screening in the general population due to their low sensitivity and specificity in detecting early primary tumor, however, they can be used clinically for the monitoring of tumor recurrence and used as prognostic factors because higher levels have been observed in advanced disease.^14^^-^^16^ With respect to controversial reports about the impact of tumor markers on the management of gastric cancer, we aimed to investigate the relationship between the preoperative tumor marker levels and different clinical aspects of gastric cancer.

## METHODS

This study was approved by local ethics committee and we got written informed consent from all subjects included in this study. Between January 2009 and January 2012, 106 consecutive patients with confirmed diagnosis of gastric cancer and 106 subjects (age and sex matched) with no malignancy as control group were included prospectively in this study. The subjects in control group were selected from the group of patients with no determined malignancy (underwent gastroscopy and / or colonoscopy, abdominal computed tomography for different reasons and having no malignant or premalignant findings in their history, physical and diagnostic examinations).

The exclusion criteria were operation history of any malignancy, having any malignancy except gastric cancer, already being a smoker, having pancreatitis. Patients’ mean age was 61.3 years (range 31-91 years); 71 were males and 35 were females.

Routine preoperative evaluation protocol of our departments consisted of clinical examination, gastroscopy and colonoscopy if indicated for clinical suspicion, abdominal computed tomography, chest radiography and computed tomography for thorax if indicated and serum levels of CEA, CA 19-9 and CA 125. We aimed to evaluate only preoperative tumor marker levels in relation with different clinical or histopathological aspects of gastric cancer. Blood samples were obtained at least one week before surgery for patient group. Serum CEA, CA 19-9 and CA 125 levels were determined with the upper limit of normal defined as 5ng/ml for CEA, 38U/ml for CA 19-9 and 35U/ml for CA 125.

The relationships between tumor markers CEA, CA 19-9 and stage of disease (with respect to American Joint Committee on Cancer, gastric cancer staging, TNM), tumor differentiation (grade), presence of ringlet cell type, presence of peritoneal carcinomatozis were investigated.

IBM^®^ The Statistical Package for Social Sciences (SPSS^®^) version 20 software was used for statistical analysis of data. All values were expressed as median (minimum- maximum). After the homogeneity tests; non – parametric tests (Mann – Whitney and Kruskal – Wallis tests) were used for statistical evaluation and p<0.05 was accepted as the level of significance.

## RESULTS

There was no statistical difference between serum CA 19-9 levels of gastric cancer patients and controls (p=0.103). On the other hand; the serum CEA and CA 125 levels were found to be significantly elevated in gastric cancer patients than in controls (p<0.001 for both) (Table-I).

The serum tumor marker levels of patients didn't show any significant difference according to either T stage or N stage of the disease (p>0.05 for all markers) (Table-II). The serum level of CEA had showed a significant elevation with the presence of distant metastasis (M1) (p=0.019) (Table-II).

The CA 19-9 and CA 125 levels had showed significant elevations where the CEA levels showed no significance with the presence of peritoneal carcinomatozis (p=0.007, p=0.018 and p=0.644 respectively) (Table-III). There was no significant difference between serum tumor marker levels of patients with respect to tumor grade or the presence of ringlet cell type of tumor cells.

## DISCUSSION

The increased levels of tumor markers such as CEA and CA 19-9 are proposed to be correlated with clinic and pathological features of gastric cancer.^17^^-^^20^ In clinical practice; the tumor markers CEA and CA 19-9 are used to assess the efficacy of adjuvant treatment as a supplementary evidence for response.^21^^,^^22^ Despite numerous reports on the usefulness of preoperative and periodic postoperative CEA measurements to predict stage,^23^ tumor progression,^24^ recurrence^25^^-^^27^ and prognosis^10^^,^^28^^,^^29^ in patients with gastric cancer, already tumor markers have limited clinical utility due to their low sensitivity and specificity.^30^^-^^32^ The positive rate of serum CEA and CA 19-9 at the initial diagnosis of gastric cancer has been reported to be 11.8%-37% ^33^^-^^37^ and 18%-45% ^22^^,^^37^ respectively. Although serum CA 19-9 levels had showed no significant difference between gastric cancer patients and controls in our study, the serum levels of CEA and CA 125 were found to be significantly elevated in patients than controls. The available data from previous studies confirm that the conventional tumor markers such as CEA and CA 19-9 don't allow diagnosis of gastric cancer with adequate sensitivity and specificity.^21^^,^^38^

**Table-I:** Tumor marker levels. Patients vs. Controls

	*Median (Minimum-Maximum)*		*p*
	*Patients*	*Controls*	
CA 19-9 (U/ml)	15 (0.8 - 4047)	10.96 (0.17 – 24.5)	0.103
CEA (ng/ml)	1.79 (0.05 – 51.43)	1.1 (0.03 – 5.01)	<0.001
CA 125 (U/ml)	11.6 (0.6 – 171.7)	6.9 (1,2 – 68.4)	<0.001

**Table-II:** Tumor marker levels with respect to TNM staging. (CA 19-9: U/ml, CEA: ng/ml, CA 125: U/ml).

	Median (Minimum – Maximum)					
	CA 19-9	p	CEA	p	CA 125	p
T1	9.85 (0.8 – 14,8)	0.188	2.07 (0.7 – 5.8)	0.795	7.95 (5.77 – 23.3)	0.737
T2	20.26 (10 – 79,9)		1.44 (0.5 – 10)		12.08 (7.6 – 54)	
T3	16.99 (0.8 – 700)		1.99 (0.64 – 14.7)		11.4 (1 – 49.6)	
T4	15.15 (1.2 – 4047)		1.81 (0.05 – 51.43)		12.4 (0.6 – 171.7)	
N0	10.61 (0.8 – 40.78)	0.067	1.7 (0.28 – 11.02)	0.802	9.85 (1-54)	0.282
N1	18.2 (6.95 – 700)		1.33 (0.33 – 10.94)		18.9 (2.5 – 171.7)	
N2	17.1 (1.2 – 3765)		1.99 (0.1 – 51.43)		13.4 (2.6 – 43.6)	
N3	14.56 (0.8 – 245)		1.93 (0.05 – 35.5)		10.15 (4.1 – 94)	
M0	15 (0.8 – 4047)	0.073	1.64 (0.05 – 35.5)	0.019	11.6 (0.6 – 171.7)	0.827
M1	15.8 (1.2 – 614.18)		4.98 (0.13 – 51.43)		11.7 (2.6 – 125.4)	

**Table-III:** Tumor marker levels with the presence of peritoneal carcinomatozis

	Peritoneal Carcinomatozis		p
	Not Present	Present	
CA 19-9 (U/ml)	14,21 (0.8 – 4047)	579,92 (14,11 – 614,18)	0.007
CEA (ng/ml)	1,81 (0.05 – 51,43)	1,67 (0.13 – 34,56)	0.644
CA 125 (U/ml)	11,3 (0.6 – 171,7)	19,7 (13,4 – 125,4)	0.018

In a previous study by Ishigami et al;^38^ it is reported that serum CEA and CA 19-9 levels are significantly and positively correlated with the depth of invasion, nodal involvement, cure possibility and distant metastasis. Our results showed that there is no significant difference correlated with depth of invasion (T stage) and lymph node involvement (N stage). However, the serum level of CEA had showed a significant elevation with the presence of distant metastasis (M1 patients). In the view of combined CEA and CA 19-9 positivity, the positivity of CEA and/or CA 19-9 may reflect biologic malignant properties such as lymphatic spread or distant metastasis.^12^^,^^14^^,^^38^ Although some studies proposed that there appears to be clinical significance in detecting CEA and CA 19-9, some studies express doubt to confirm this way of management.^10^^-^^12^^,^^39^ The positivity of CEA and CA 19-9 was significantly correlated with TNM stage, depth of invasion and lymph node metastasis in a more recent study.^40^ Despite the numerous reports on the usefulness of preoperative and postoperative CEA measurements to predict stage,^23^ tumor progression,^24^ recurrence^25^ or prognosis;^28^ there is no agreement as to what kinetics of change is likely to be significant and over what period of time such a change should be maintained for significance.^41^ The positivity of CEA in the presence of distant metastasis may be explained with the direct role of CEA by acting like an adhesion molecule in invasion and metastasis so the cancer cells producing CEA have more chance of metastasis.^42^^,^^43^

Peritoneal dissemination is frequent and life threatening form of metastasis and recurrence in patients with gastric cancer. Intraperitoneal chemotherapy has been shown to have a considerable positive effect on peritoneal dissemination. Although the systemic chemotherapy has been shown to prolong the survival of gastric cancer patients, there is a lack of complete success.^44^^-^^46^ Serum CA 125 levels are known to be elevated in peritoneal inflammation and in carcinomatozis^47^^,^^48^ and a significant relationship between CA 125 and gastric cancer with peritoneal dissemination has been reported.^49^^-^^51^ The CA 19-9 and CA 125 levels had showed significant elevations with the presence of peritoneal carcinomatozis in our study. The presence of ascites, level of ascites and the degree of peritoneal metastasis are not correlated with CEA and CA 19-9; however they have a significant correlation with CA 125 positivity.^44^ The serum level of CA 125 was reported to be more sensitive in combination with other tumor markers such as CA 19-9 for peritoneal dissemination.^44^

Some authors have tried to explain the low sensitivity of tumor markers in their studies in terms of the histology of the tumor, with the diffuse type of gastric cancer presenting the lowest positivity rate of the tumor markers. On the other hand, some previous reports made this hypothesis controversial with the higher positivity rate of CEA, CA 72-4 and CA 19-9 in the diffuse type of gastric cancer.^38^^,^^52^ Our results showed that there was no significant difference between serum tumor marker levels of patients with respect to tumor grade or the presence of ringlet cell type of tumor cells. It is also shown that no correlation between tumor marker levels and the histology of gastric cancer.^14^

As a conclusion; there seems to be many controversial and conflicting reports about the relationship of tumor markers and the clinical properties of gastric cancer. Our results also showed that there is a limited clinical benefit of preoperative tumor marker measurements in gastric cancer such as estimation of peritoneal dissemination. The various types of biological behaviors of gastric cancer need further studies on molecular basis of tumor cells and tumor markers.

## Author Contributions:

Erdal Polat, Uğur Duman and Mustafa Duman; designed the study, had contribution for data acquisition, analyzed data; wrote, revised and approved the main text. Kivanc Derya Peker, Cebrail Akyuz, Necdet Fatih Yasar, Orhan Uzun and Sabiye Akbulut had contribution for acquisition of patients' data, wrote and approved the manuscript. Erdal Birol Bostanci and Sinan Yol; designed the study, revised and finally approved the manuscript.

## References

[1] Parkin DM, Bray F, Ferlay J, Pisani P (2005). Global cancer statistics, 2002. CA Cancer J Clin.

[2] Lochhead P, El-Omar EM (2008). Gastric cancer. Br Med Bull.

[3] Jemal A, Bray F, Center MM, Ferlay J, Ward E, Forman D (2011). Global cancer statistics. CA Cancer J Clin.

[4] Brenner H, Rothenbacher D, Arndt V (2009). Epidemiology of stomach cancer. Methods Mol Biol.

[5] Liakakos T, Roukos DH ( 2008). More controversy than ever - challenges and promises towards personalized treatment of gastric cancer. Ann Surg Oncol.

[6] McCulloch P, Nita ME, Kazi H (2004). Extended versus limited lymph nodes dissection technique for adenocarcinoma of the stomach. Gama-Rodrigues J.

[7] Cunningham D, Allum WH, Stenning SP, Thompson JN, Van de Velde CJ, Nicolson M (2006). Perioperative chemotherapy versus surgery alone for resectable gastroesophageal cancer. N Engl J Med.

[8] Kwee RM, Kwee TC (2009). Imaging in assessing lymph node status in gastric cancer. Gastric Cancer.

[9] Webb A, Scott-Mackie P, Cunningham D, Norman A, Andreyev J, O'Brien M (1996). The prognostic value of serum and immunohistochemical tumour markers in advanced gastric cancer. Eur J Cancer.

[10] Nakane Y, Okamura S, Akehira K, Boku T, Okusa T, Tanaka K (1994). Correlation of preoperative carcinoembryonic antigen levels and prognosis of gastric cancer patients. Cancer.

[11] Kodera Y, Yamamura Y, Torii A, Uesaka K, Hirai T, Yasui K (1996). The prognostic value of preoperative serum levels of CEA and CA19-9 in patients with gastric cancer. Am J Gastroenterol.

[12] Ikeda Y, Oomori H, Koyanagi N, Mori M, Kamakura T, Minagawa S (1995). Prognostic value of combination assays for CEA and CA 19-9 in gastric cancer. Oncology.

[13] Yamao T, Kai S, Kazami A, Koizumi K, Handa T, Takemoto N (1999). Tumor markers CEA, CA19-9 and CA125 in monitoring of response to systemic chemotherapy in patients with advanced gastric cancer. Jpn J Clin Oncol.

[14] Marrelli D, Roviello F, De Stefano A, Farnetani M, Garosi L, Messano A (1999). Prognostic significance of CEA, CA 19-9 and CA 72-4 preoperative serum levels in gastric carcinoma. Oncology.

[15] Mattar R, Alves de Andrade CR, DiFavero GM, Gama-Rodrigues JJ, Laudanna AA (2002). Preoperative serum levels of CA 72-4, CEA, CA 19-9, and alpha-fetoprotein in patients with gastric cancer. Rev Hosp Clin Fac Med Sao Paulo.

[16] Ikeguchi M, Katano K, Saitou H, Tsujitani S, Maeta M, Kaibara N (1997). Pre-operative serum levels of CA72-4 in patients with gastric adenocarcinoma. Hepatogastroenterology.

[17] Ilhan N, Ilhan N, Ilhan Y, Akbulut H, Kucuksu M (2004). C-reactive protein, procalcitonin, interleukin-6, vascular endothelial growth factor and oxidative metabolites in diagnosis of infection and staging in patients with gastric cancer. World J Gastroenterol.

[18] Ikeguchi M, Hatada T, Yamamoto M, Miyake T, Matsunaga T, Fukumoto Y (2009). Serum interleukin-6 and -10 levels in patients with gastric cancer. Gastric Cancer.

[19] Mroczko B, Groblewska M, Lukaszewicz-Zajac M, Bandurski R, Kedra B, Szmitkowski M (2009). Pre-treatment serum and plasma levels of matrix metalloproteinase 9 (MMP-9) and tissue inhibitor of matrix metalloproteinases 1 (TIMP-1) in gastric cancer patients. Clin Chem Lab Med.

[20] Mroczko B, Wereszczynska-Siemiatkowska U, Groblewska M, Lukaszewicz M, Szmitkowski M, Gryko M (2006). The diagnostic value of hematopoietic cytokines measurement in the sera of gastric cancer and gastric ulcer patients. Clin Chim Acta.

[21] Kochi M, Fujii M, Kanamori N, Kaiga T, Kawakami T, Aizaki K (2000). Evaluation of serum CEA and CA19-9 levels as prognostic factors in patients with gastric cancer. Gastric Cancer.

[22] Takahashi Y, Takeuchi T, Sakamoto J, Touge T, Mai M, Ohkura H (2003). The usefulness of CEA and/or CA19-9 in monitoring for recurrence in gastric cancer patients: a prospective clinical study. Gastric Cancer.

[23] Staab HJ, Anderer FA, Brummendorf T, Hornung A, Fischer R (1982). Prognostic value of preoperative serum CEA level compared to clinical staging: II. Stomach cancer. Br J Cancer.

[24] Dittrich C, Jakesz R, Havelec L, Lenzhofer R, Breyer S, Moser K (1985). Carcinoembryonic antigen (CEA) plasma level determination in the management of gastric cancer patients. Cancer Detect Prev.

[25] Tamada R, Hiramoto Y, Tsujitani S, Nozuka T, Okamura T, Masuda H (1985). Serum CEA levels facilitate detection of recurrences of cancer in patients after gastrectomy. Jpn J Surg.

[26] Shimizu N, Wakatsuki T, Murakami A, Yoshioka H, Hamazoe R, Kanayama H (1987). Carcinoembryonic antigen in gastric cancer patients. Oncology.

[27] Maehara Y, Sugimachi K, Akagi M, Kakegawa T, Shimazu H, Tomita M (1990). Serum carcinoembryonic antigen level increases correlate with tumor progression in patients with differentiated gastric carcinoma following noncurative resection. Cancer Res.

[28] Maehara Y, Kusumoto T, Takahashi I, Kakeji Y, Baba H, Akazawa K ( 1994). Predictive value of preoperative carcinoembryonic antigen levels for the prognosis of patients with well-differentiated gastric cancer. A multivariate analysis. Oncology.

[29] Sakamoto J, Nakazato H, Teramukai S, Ohashi Y, Takahashi Y, Mai M (1996). Association between preoperative plasma CEA levels and the prognosis of gastric cancer following curative resection. Tumor Marker Committee, Japanese Foundation for Multidisciplinary Treatment of Cancer, Tokyo, Japan. Surg Oncol.

[30] Heptner G, Domschke S, Domschke W (1989). Comparison of CA 72-4 with CA 19-9 and carcinoembryonic antigen in the serodiagnostics of gastrointestinal malignancies. Scand J Gastroenterol.

[31] Kornek G, Depisch D, Temsch EM, Scheithauer W (1991). Comparative analysis of cancer-associated antigen CA-195, CA 19-9 and carcinoembryonic antigen in diagnosis, follow-up and monitoring of response to chemotherapy in patients with gastrointestinal cancer. J Cancer Res Clin Oncol.

[32] Staab HJ, Anderer FA, Brummendorf T, Fischer R (1983). Prognostic significance of preoperative carcinoembryonic antigen in stomach and colorectal cancer. Cancer Detect Prev.

[33] Janssen CW Jr, Orjasaeter H (1986). Carcinoembryonic antigen in patients with gastric carcinoma. Eur J Surg Oncol.

[34] Koga T, Kano T, Souda K, Oka N, Inokuchi K (1987). The clinical usefulness of preoperative CEA determination in gastric cancer. Jpn J Surg.

[35] Kim YH, Ajani JA, Ota DM, Lynch P, Roth JA (1995). Value of serial carcinoembryonic antigen levels in patients with resectable adenocarcinoma of the esophagus and stomach. Cancer.

[36] Nishiyama M, Takashima I, Tanaka T, Yoshida K, Toge T, Nagata N (1995). Carcinoembryonic antigen levels in the peritoneal cavity: useful guide to peritoneal recurrence and prognosis for gastric cancer. World J Surg.

[37] Duraker N, Celik AN (2001). The prognostic significance of preoperative serum CA 19-9 in patients with resectable gastric carcinoma: comparison with CEA. J Surg Oncol.

[38] Ishigami S, Natsugoe S, Hokita S, Che X, Tokuda K, Nakajo A (2001). Clinical importance of preoperative carcinoembryonic antigen and carbohydrate antigen 19-9 levels in gastric cancer. J Clin Gastroenterol.

[39] Mori M, Sakaguchi H, Akazawa K, Tsuneyoshi M, Sueishi K, Sugimachi K ( 1995). Correlation between metastatic site, histological type, and serum tumor markers of gastric carcinoma. Hum Pathol.

[40] Qiu MZ, Lin JZ, Wang ZQ, Wang FH, Pan ZZ, Luo HY (2009). Cutoff value of carcinoembryonic antigen and carbohydrate antigen 19-9 elevation levels for monitoring recurrence in patients with resectable gastric adenocarcinoma. Int J Biol Markers.

[41] Guadagni F, Roselli M, Amato T, Cosimelli M, Perri P, Casale V (1992). CA 72-4 measurement of tumor-associated glycoprotein 72 (TAG-72) as a serum marker in the management of gastric carcinoma. Cancer Res.

[42] Benchimol S, Fuks A, Jothy S, Beauchemin N, Shirota K, Stanners CP (1989). Carcinoembryonic antigen, a human tumor marker, functions as an intercellular adhesion molecule. Cell.

[43] Grimm T, Johnson JP: Ectopic expression of carcinoembryonic antigen by a melanoma cell leads to changes in the transcription of two additional cell adhesion molecules (1995). Cancer Res.

[44] Emoto S, Ishigami H, Yamashita H, Yamaguchi H, Kaisaki S, Kitayama J (2012). Clinical significance of CA125 and CA72-4 in gastric cancer with peritoneal dissemination. Gastric Cancer.

[45] Ishigami H, Kitayama J, Kaisaki S, Hidemura A, Kato M, Otani K (2010). Phase II study of weekly intravenous and intraperitoneal paclitaxel combined with S-1 for advanced gastric cancer with peritoneal metastasis. Ann Oncol.

[46] Kodera Y, Ito Y, Ito S, Ohashi N, Mochizuki Y, Yamamura Y (2007). Intraperitoneal paclitaxel: a possible impact of regional delivery for prevention of peritoneal carcinomatosis in patients with gastric carcinoma. Hepatogastroenterology.

[47] Eisenhauer EA, Therasse P, Bogaerts J, Schwartz LH, Sargent D, Ford R (2009). New response evaluation criteria in solid tumours: revised RECIST guideline (version 1.1). Eur J Cancer.

[48] Rustin GJ, Vergote I, Eisenhauer E, Pujade-Lauraine E, Quinn M, Thigpen T (2011). Definitions for response and progression in ovarian cancer clinical trials incorporating RECIST 1.1 and CA 125 agreed by the Gynecological Cancer Intergroup (GCIG). Int J Gynecol Cancer.

[49] Nakata B, Hirakawa YSCK, Kato Y, Yamashita Y, Maeda K, Onoda N (1998). Serum CA 125 level as a predictor of peritoneal dissemination in patients with gastric carcinoma. Cancer.

[50] Hwang GI, Yoo CH, Sohn BH, Shin JH, Park YL, Kim HD (2004). Predictive value of preoperative serum CEA, CA19-9 and CA125 levels for peritoneal metastasis in patients with gastric carcinoma. Cancer Res Treat.

[51] Fujimura T, Kinami S, Ninomiya I, Kitagawa H, Fushida S, Nishimura G (2002). Diagnostic laparoscopy, serum CA125, and peritoneal metastasis in gastric cancer. Endoscopy.

[52] Kodama I, Koufuji K, Kawabata S, Tetsu S, Tsuji Y, Takeda J (1995). The clinical efficacy of CA 72-4 as serum marker for gastric cancer in comparison with CA19-9 and CEA. Int Surg.

